# Clinical Outcomes of Radical Surgery and Antimicrobial Agents in Vascular Pythiosis: A Multicenter Prospective Study

**DOI:** 10.3390/jof7020114

**Published:** 2021-02-04

**Authors:** Pattama Torvorapanit, Nipat Chuleerarux, Rongpong Plongla, Navaporn Worasilchai, Kasama Manothummetha, Achitpol Thongkam, Nattapong Langsiri, Jaruwan Diewsurin, Prasopchai Kongsakpaisan, Ratiporn Bansong, Nuttapon Susaengrat, Watchara Wattanasoontornsakul, Ariya Chindamporn, Nitipong Permpalung

**Affiliations:** 1 Faculty of Medicine, Chulalongkorn University, Bangkok 10330, Thailand; pigdapaii@yahoo.com (P.T.); jade.negi.nipat@gmail.com (N.C.); rongpong@hotmail.com (R.P.); kasamark.m@gmail.com (K.M.); tachitpol@docchula.com (A.T.); nutleng1150@gmail.com (N.L.); drariya@gmail.com (A.C.); 2 King Chulalongkorn Memorial Hospital, Thai Red Cross Society, Bangkok 10330, Thailand; 3 Faculty of Allied Health Sciences, Chulalongkorn University, Bangkok 10330, Thailand; navaporn.w@chula.ac.th; 4 Buddhachinaraj Hospital, Phitsanulok 65000, Thailand; Kratae012@hotmail.com; 5 Nakhon Pathom Hospital, Nakhon Pathom 73000, Thailand; prasopchai099@gmail.com; 6 Sunpasithiprasong Hospital, Ubon Ratchathani 34000, Thailand; a_om_07@hotmail.com; 7 Khonkaen Hospital, Khonkean 40000, Thailand; nuttapon_md@hotmail.com; 8 Maharat Nakhon Ratchasima Hospital, Nakhon Ratchasima 30000, Thailand; watchnhr@gmail.com; 9 Johns Hopkins University School of Medicine, Baltimore, MD 21205, USA

**Keywords:** human pythiosis, *P. insidiosum*, vascular pythiosis, β-d-glucan, ESR, CRP

## Abstract

Vascular pythiosis is a rare, neglected, life-threatening disease with mortality of 100% in patients with incomplete surgical resection or patients with persistently elevated serum β-d-glucan (BDG). The study was conducted to understand the clinical outcomes of new treatment protocols and potential use of erythrocyte sedimentation rate (ESR) and c-reactive protein (CRP) as alternative monitoring tools, given recent favorable minimum inhibitory concentrations (MICs) of antibacterial agents and prohibitive cost of serum BDG in Thailand. A prospective cohort study of patients with vascular pythiosis was conducted between February 2019 and August 2020. After diagnosis, patients were followed at 0.5, 1, 1.5, 3, and 6 months. Descriptive statistics, Spearman’s correlation coefficient, and general linear model for longitudinal data were used. Amongst the cohort of ten vascular pythiosis patients, four had residual disease after surgery. Among four with residual disease, one developed disseminated disease and died, one developed relapse disease requiring surgery, and two were successfully managed with antimicrobial agents. The spearman’s correlation coefficients between BDG and ESR, and between BDG and CRP in patients without relapse or disseminated disease were 0.65 and 0.60, respectively. Tetracyclines and macrolides had most favorable minimum inhibitory concentrations and synergistic effects were observed in combinations of these two antibiotic classes. Adjunctive use of azithromycin and doxycycline preliminarily improved survival in vascular pythiosis patients with residual disease. Further studies are needed to understand the trends of ESR and CRP in this population.

## 1. Introduction

*Pythium insidiosum,* an aquatic fungal-like pathogen, causes pythiosis in humans. Human pythiosis was first described in a Thai thalassemia patient in 1988 [1]. The clinical presentations are classified into four groups: vascular, ocular, subcutaneous/cutaneous, and disseminated infection [2]. Human pythiosis has also been reported from the United States of America, Canada, Brazil, Frances, Israel, India, China, and Australia [3,4,5,6,7,8,9]. In Thailand, vascular pythiosis is the most common form and it is associated with a mortality rate of 10–40%, depending on the rate of successful surgery [10,11,12,13]. Unfortunately, mortality was up to 100% in patients without complete surgical resection regardless of other adjunctive therapy [10,11,12,13]. Because of its low prevalence, a high index of suspicion is crucial in identifying disease, particularly in those with risk factors who present with arterial insufficiency or chronic lower extremity wounds [14]. Risk factors often include occupation in agriculture and/or underlying hemoglobinopathy, including thalassemia, in patients presenting from an endemic region [2,10,13,14]. The proposed mechanisms of infection in patients with hemoglobinopathy are interference of phagocytic activities of macrophages and neutrophils from iron overload, lower interferon gamma (IFN-γ) production in Thalassemia patients, and intrinsic virulence of *P. insidiosum* by carrying a gene encoding ferrochelatase [15,16,17].

Treatment strategies remain challenging, and radical surgery is the mainstay. Itraconazole plus terbinafine were formerly recommended as the backbone antimicrobial therapy along with radical surgery. This regimen was largely based on in vitro susceptibility data from Brazilian *P. insidiosum* isolates [18,19,20]. However, Thai *P. insidiosum* isolates had unfavorable antifungal minimum inhibitory concentrations (MICs) and antifungal agents did not improve survival outcomes in patients infected with *P. insidiosum* [10,13,21]. Recent study from the King Chulalongkorn Memorial Hospital (KCMH) pythiosis research group showed that Thai *P. insidiosum* isolates were susceptible to azithromycin and doxycycline [22,23]. Hence, the antimicrobial regimens under the KCMH research protocols were transitioned to itraconazole plus azithromycin; subsequently, doxycycline was added in the treatment protocol due to evidence of a synergistic effect with azithromycin and doxycycline in combination against Thai *P. insidiosum* isolates [22,23].

Early disease recognition and definitive surgical resection are associated with reduced mortality [10,13]. However, patients can have residual infection despite negative surgical margins. These patients typically demonstrate persistent elevation of the serum beta-d-glucan (BDG) postoperatively [13]. Therefore, serum BDG has been integrated into the KCMH vascular pythiosis research protocols for monitoring disease. Widespread use of serum BDG monitoring is limited in Thailand outside research protocols because the cost is prohibitive and the assay availability is limited to KCMH. This study was conducted to describe clinical outcomes of new treatment protocols incorporating surrogate markers, erythrocyte sedimentation rate (ESR) and c-reactive protein (CRP), as potential inexpensive monitoring tools which are more easily and globally accessible.

## 2. Patients and Methods

### 2.1. Patients and Study Design

This is a multicenter, prospective cohort study of vascular pythiosis patients who were treated according to the KCMH research treatment protocols with a combination of radical surgery and antimicrobial agents from February 2019 to August 2020. Given evidence of antimicrobial synergy between tetracyclines and macrolides, doxycycline was added into the antimicrobial treatment regimen starting in April 2019. Patients were eligible for the study if at least two pythiosis diagnostic criteria were met: (1) clinical presentation consistent with vascular pythiosis; (2) successful isolation of *P. insidiosum* with zoospore induction [24]; (3) positive *P. insidiosum*-specific antibody (*Pi*-Ab) by enzyme-linked immunosorbent assay (ELISA) [25,26]; (4) positive tissue polymerase chain reaction (PCR) for *P. insidiosum* using internal transcribed spacer or cytochrome oxidase II (PCR-ITS/*COX2*) primers [27,28,29]; (5) presence of sparsely septate hyphae consistent with *P. insidiosum* in tissue pathology. During the study period, clinical data and blood specimens were obtained at diagnosis, 0.5, 1, 1.5, 3, and 6 months. Subsequent clinic visits after 6 months were per clinical discretion if there was concern for persistent disease or relapsed infection. The study was approved by the Institutional Review Board at all study sites and informed consent was obtained from all patients.

### 2.2. Beta-d-Glucan, Erythrocyte Sedimentation Rate, and C-Reactive Protein

Serum BDG was evaluated using the Fungitell assay (Associates of Cape Cod, Inc., Falmouth, MA, USA) according to the manufacturer’s instruction. Samples with BDG levels < 31 pg/mL and >500 pg/mL although outside the Fungitell assay range (31–500 pg/mL) were statistically analyzed as 31 pg/mL and 500 pg/mL, respectively [30,31]. Serum ESR was tested by an automated analyzer (JOKOH Monitor-20, Tokyo, Japan) using an infrared barrier method. Normal range is 0–15 mm/h for males and 0–28 mm/h for females. Serum CRP was measured via immunoturbidimetric assay on a clinical chemistry analyzer (Abbott Alinity ci-series, Chicago, IL, USA, normal range 0–5 mg/L).

### 2.3. In Vitro Susceptibility

*P. insidiosum* was successfully isolated from eight patients. Broth microdilution was performed according to Clinical and Laboratory Standards Institute (CLSI) M38, third edition, Guidelines for Filamentous Fungi [32]. In vitro susceptibility was subsequently performed according to published methods and all assays were performed in triplicate [22,33,34,35]. We induced zoospores and diluted the inoculum (2 × 10^3^ to 3 × 10^3^ zoospores/mL) with Roswell Park Memorial Institute (RPMI) 1640 broth, pH 7.0 (with glucose and L-glutamine). The MICs were determined by direct visual observation of 100% inhibition of mycelium growth after incubation for 48 h. Eight *P. insidiosum* isolates were tested against eight antibacterial and four antifungal agents. The in vitro synergy of the two antimicrobial classes was determined according to the checkerboard technique [22,23]. The results were interpreted as synergism when the fractional inhibitory concentration index (FICI) ≤ 0.5; indifference when 0.5 < FICI < 4.0; antagonism when FICI > 4.0.

### 2.4. Statistical Analyses

The statistical analyses were conducted by SAS University edition (SAS Institute, Cary, NC, USA). Descriptive statistics were used to describe serum BDG, ESR, and CRP at each time point. General linear model for longitudinal data were used for the single group repeated measure design to understand the effect of time on the mean response of serum BDG, ESR, and CRP. Spearman’s correlation coefficient was used to evaluate correlation among serum BDG, ESR, and CRP. The general linear model for longitudinal data and Spearman’s correlation coefficient were only performed in patients without relapse or disseminated disease in this study given differences in the nature of the diseases.

## 3. Results

### 3.1. Patient Characteristics and Treatment Modalities

Ten patients met the diagnostic inclusion criteria and were enrolled into the study. Baseline patient characteristics, clinical presentation, and treatment modalities are summarized in Table 1. All patients had underlying thalassemia. Duration from the first medical encounter to radical surgery ranged from 1 to 60 days. Radical surgery was performed on all patients; however, four patients had residual disease after the first surgery (three without negative surgical margins and one with relapsed disease). Among three patients without negative surgical margins (cases 1, 2, and 9), one (case 9) was diagnosed with disseminated disease at the time of diagnosis and died 5 months after diagnosis. Case 1 and 2 were successfully managed with antimicrobial agents and did not require additional surgery. Case 8, with negative surgical margins, required a second surgery given clinical concern for relapsed disease and remained well through the end of the study period. All patients tolerated antimicrobial agents well without side effects during the study period.

### 3.2. Serum Beta-d-Glucan, Erythrocyte Sedimentation Rate and C-Reactive Protein

**Figure 1:**
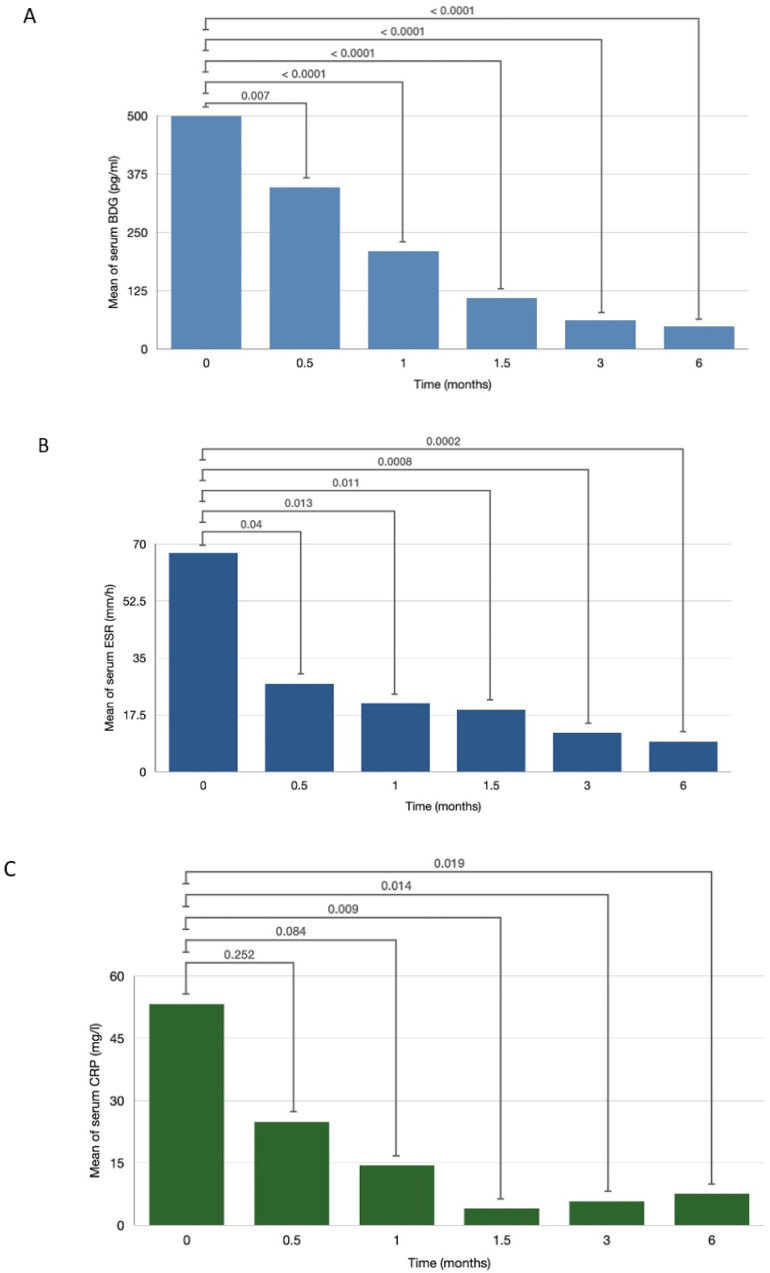
Means of serum β-d-glucan (BDG) (**A**), serum erythrocyte sedimentation rate (ESR) (**B**), and serum C-reactive protein (CRP) (**C**) across different time points.

At the time of diagnosis, all patients had highly elevated serum BDG (>500 pg/mL), ESR (>60 mm/h) and CRP (>50 mg/L). In patients without relapsed or disseminated disease, the mean of serum BDG significantly decreased at 0.5 months after treatment initiation (347 vs. 500 pg/mL, *p* = 0.007). Mean serum BDG continued to decrease over time and was lower than the positive cut-off value (<80 pg/mL) by 3 months (Figure 1A). Similarly, among patients without relapsed or disseminated disease, the mean serum ESR significantly decreased at 0.5 months after treatment initiation (27.13 vs. 67.29 mm/h, *p* = 0.04). Mean serum ESR decreased linearly over time and had also normalized by 3 months (Figure 1B). The mean serum CRP also declined following treatment initiation, demonstrating a statistically significant decrease at 1.5 months (3.99 vs. 53.28 mg/L, *p* = 0.009) (Figure 1C). Spearman’s correlation coefficients between BDG and ESR, and between BDG and CRP were 0.65 and 0.60, respectively.

**Figure 2:**
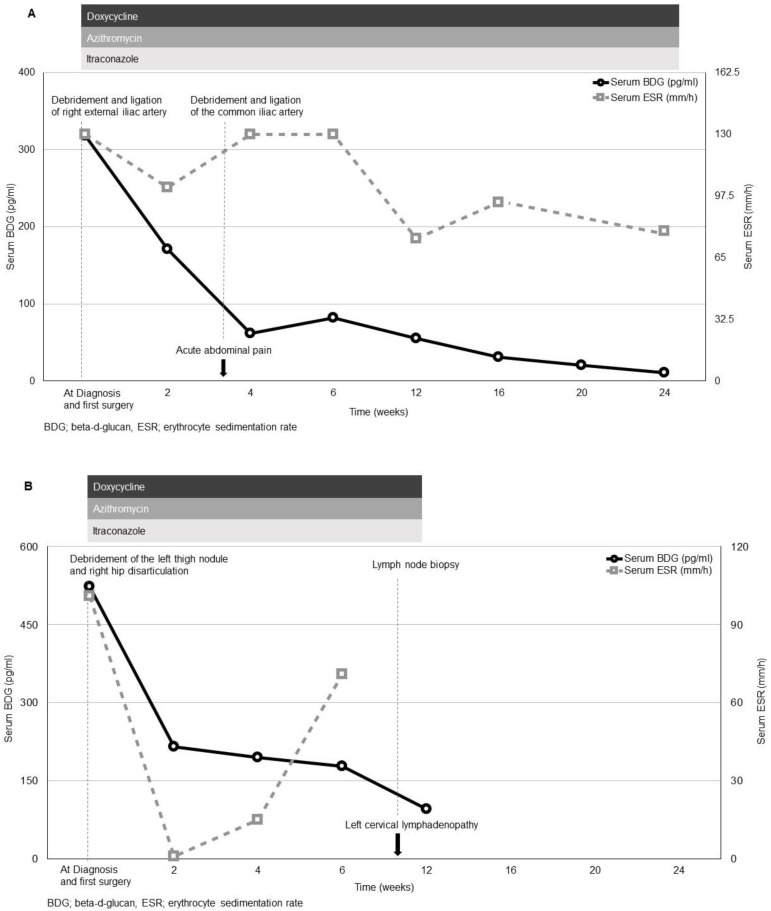
Clinical course and treatment modalities of relapsed disease (**A**) and disseminated disease (**B**).

Case 8 with relapsed disease and case 9 with disseminated disease were separately described given their distinct features. In case 8, serum BDG, ESR, and CRP initially declined after the first surgery. However, the patient developed acute abdominal pain and a computed tomography angiography showed thickening wall of the right common iliac artery before the scheduled visit at 1 month. The patient underwent emergent debridement and ligation of the common iliac artery. The pathology did not reveal evidence of fungal disease. The patient’s ESR and CRP were checked at 1 month per study protocol and they were elevated after the surgery. After the second surgery, the patient did well clinically and patient’s serum BDG and CRP normalized at 6 months after diagnosis. However, the patient’s ESR remained elevated throughout the study period (Figure 2A).

**Figure 3:**
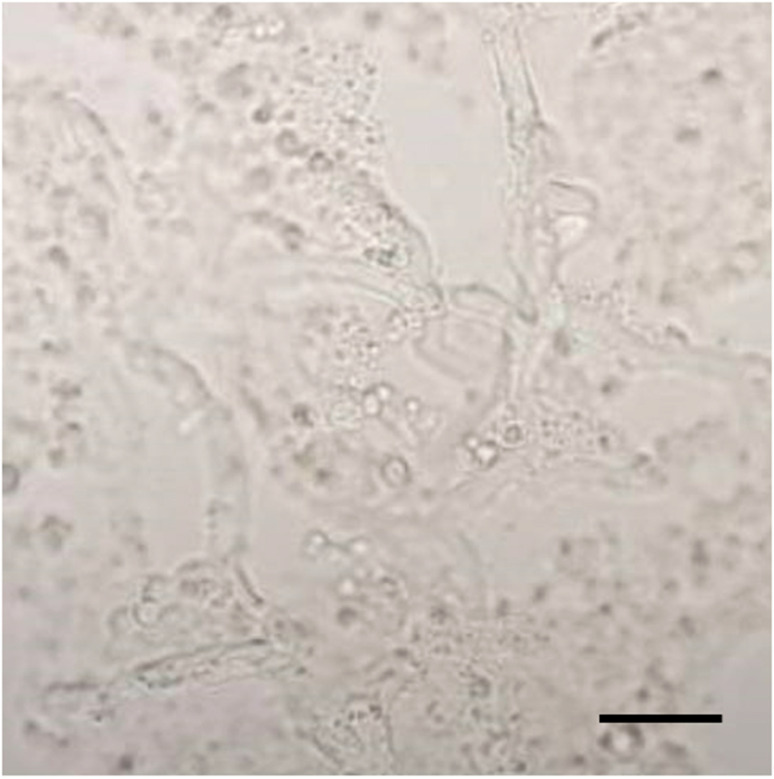
Direct examination of the vascular tissue by using potassium hydroxide preparation showed sparsely septate hyphae (×400; the bar scale is 20 µm).

In case 9, serum BDG, ESR, and CRP initially declined after initial treatment (right hip disarticulation, wide excision of the subcutaneous nodule at the left thigh and antimicrobial therapy with itraconazole, azithromycin and doxycycline). The patient was diagnosed with disseminated pythiosis given presence of fungal elements from the right lower extremity vessels and from the skin lesion on the left thigh. Microscopic examination of the vascular tissue by using 10% potassium hydroxide preparation showed scarcely septate hyphae consistent with *P. insidiosum* (Figure 3). Computed tomography angiography at the time of diagnosis did not reveal disease involvement of the left lower extremity vessels. Prognosis was thoroughly discussed, and the patient elected to continue only medical therapy. Subsequent serum BDG, ESR, and CRP remained significantly elevated. Unfortunately, the patient developed left neck swelling and the lymph node biopsy showed fungal element consistent with *P. insidiosum.* Case 9 died at 5 months after initial diagnosis (Figure 2B).

### 3.3. In Vitro Susceptibility

The in vitro susceptibility results are summarized in Table 2. Among eight antibacterial classes, tetracyclines (doxycycline, minocycline, and tigecycline) and macrolides (azithromycin and clarithromycin) had favorable MICs, compared to other classes. Synergistic effects between tetracyclines and macrolides were observed in all *P. insidiosum* isolates. Among seven antifungal agents, itraconazole showed favorable MICs, compared to other antifungal agents. No synergistic effects were observed with combinations of voriconazole/terbinafine and itraconazole/terbinafine. The *P. insidiosum* isolate from case 9 with disseminated disease had higher MIC values (doxycycline MIC 4, azithromycin MIC 2, itraconazole MIC 4, and voriconazole MIC 8).

## 4. Discussion

We describe the first multicenter, prospective study to preliminarily report the potential clinical benefit of antibacterial agents and the use of ESR and CRP as monitoring tools in vascular pythiosis. All ten patients underwent radical surgery. Although, negative surgical margins were successfully obtained in seven patients, one death occurred in a patient with disseminated pythiosis. Despite lack of a control group and small sample size, the findings are encouraging. Adjunctive itraconazole, azithromycin, and doxycycline along with close monitoring of serum BDG for treatment response and early relapse detection likely provides survival benefit, particularly in those with residual disease. A multicenter, open-label, single arm clinical trial is being conducted in Thailand to fully understand the efficacy of the new treatment protocol and the study will be completed in 2023 (Thai Clinical Trial Registry number: TCTR20191217006).

In our previous study, we have found that patients with persistently elevated serum BDG after surgery died within 3 months and this phenomenon occurred in patients with incomplete surgical resection or patients with relapsed disease regardless of antifungal therapy [13]. Interestingly, only the patient with disseminated pythiosis had persistently elevated serum BDG while the other three patients with residual disease (cases 1, 2, and 8) did well postoperatively. Based on our in vitro susceptibility results, tetracyclines and macrolides had 10–100 times lower MICs than antifungal agents in each *P. insidiosum* isolate and synergistic effects were observed in combinations of tetracyclines and macrolides in all *P. insidiosum* isolates. Similar to previous Thai *P. insidiosum* in vitro susceptibility results, no synergistic effects were observed with combinations of itraconazole/terbinafine or voriconazole/terbinafine [10,13,21]. We observed that the *P. insidiosum* isolate from the patient with disseminated disease had elevated MICs in all classes of antimicrobial agents which may explain why the patient did not respond to medical treatment. However, it is important to note that standardized MIC interpretations for *P. insidiosum* have not been established.

ESR and CRP are non-specific inflammatory markers used in monitoring various infections such as infective endocarditis and osteomyelitis [36,37,38,39,40]. ESR and CRP are inexpensive and accessible nationwide in Thailand. Given their established use in monitoring endovascular infections, we investigated their potential role in monitoring vascular pythiosis. Serum BDG demonstrated plausible correlation with ESR and CRP levels across time. We expect that the correlation coefficients would be higher in the larger study. Similar to our previous study of serial serum BDG monitoring [13], we believe serum ESR and CRP trends predict response to therapy.

Our main limitation is the small sample size in this rare disease. The correlation was only calculated from patients without relapsed or disseminated disease and no control arm was used. In clinical practice, ESR and CRP can be challenging to trend especially owing to inter-laboratory variation.

In summary, azithromycin and doxycycline should be considered in addition to surgery and antifungal agent(s) in vascular pythiosis treatment; however, antimicrobial regimens should be adjusted based on local susceptibility data. ESR and CRP demonstrate a preliminarily correlation with serum BDG in response to vascular pythiosis treatment in patients without relapse or disseminated disease. Further studies are needed to evaluate efficacy of treatment modalities and to understand patterns of ESR and CRP in pythiosis patients, particularly with relapse or disseminated disease.

## Figures and Tables

**Table 1:** Patient characteristics and treatment modalities.

Case	Age (Years) /Sex	Occupation	Clinical Presentations	Arterial Involvement	Surgery	Duration from First Medical Visit to Surgery (Days)	Free Surgical Margins Achievement	Antimicrobial Agents	Follow-Up Duration (Months)
1	53/M	Farmer	Left leg pain 2 weeks	Left popliteal artery	Left AKA	5	No	Itraconazole Azithromycin	6
2	52/M	Driver	Right leg pain, groin mass, cold skin, numbness 3 days	Right common iliac artery	Right AKA	5	No	Itraconazole Azithromycin Doxycycline	6
3	69/M	Farmer	Left leg pain 2 weeks	Left tibial artery and left popliteal artery	Left AKA	5	Yes	Itraconazole Azithromycin	6
4	34/M	Merchant	Right ankle swelling and tenderness 2 days	Right popliteal artery	Right BKA	25	Yes	Itraconazole Azithromycin	8
5	50/F	Farmer	Right leg chronic wound 2 months	Right popliteal artery	Right BKA	21	Yes	Itraconazole Azithromycin	8
6	53/M	Farmer	Left leg pain 1 day	Left femoral artery	Left AKA	1	Yes	Itraconazole Azithromycin Doxycycline	6
7	49/M	Security guard	Left leg chronic wound 3 years	Cutaneous vessels	Left wide excision	11	Yes	Itraconazole Azithromycin Doxycycline	6
8	45/M	Merchant	Right leg pain and chronic wound 2 months	Right femoral artery	Ligation of right external iliac artery	6	Yes	Itraconazole Azithromycin Doxycycline	6
9	51/M	Potter	Right leg pain 2 months	Right femoral artery	Right hip disarticulation	60	No	Itraconazole Azithromycin Doxycycline	5
10	53/M	Farmer	Left leg pain 2 months	Left anterior tibial artery	Left AKA	7	Yes	Itraconazole Azithromycin Doxycycline	6

AKA: above knee amputation; BKA: below knee amputation; F: female; M: male.

**Table 2:** In vitro susceptibility results of *Pythium insidiosum* isolates.

		Minimum Inhibitory Concentrations (mg/L)							
		Case 1	Case 2	Case 3	Case 5	Case 6	Case 8	Case 9	Case 10
Tetracyclines	Doxycycline	4	1	4	2	2	4	4	2
	Minocycline	1	1	0.5	1	0.25	1	1	2
	Tigecycline	1	1	0.5	1	1	1	2	1
Macrolides	Azithromycin	4	2	2	2	2	2	2	4
	Clarithromycin	2	1	1	1	0.125	1	2	1
Beta-lactams	Cefazolin	>32	>32	>32	>32	>32	>32	>32	>32
	ceftriaxone	>32	>32	>32	>32	>32	>32	>32	>32
	Ceftazidime	>32	>32	>32	>32	>32	>32	>32	>32
	Meropenem	32	32	>32	32	32	>32	>32	>32
Oxazolidinone	Linezolid	4	8	4	8	4	4	8	4
Glycopeptide	Vancomycin	>32	>32	>32	>32	>32	>32	>32	>32
Aminoglycosides	Amikacin	>32	>32	>32	>32	>32	>32	>32	>32
	Gentamicin	32	>32	16	32	>32	>32	>32	>32
	Neomycin	32	>32	>32	>32	>32	>32	>32	>32
	Streptomycin	32	>32	>32	32	32	16	>32	32
	Tobramycin	>32	>32	>32	>32	>32	>32	>32	>32
Quinolones	Ciprofloxacin	>32	>32	>32	>32	>32	>32	>32	>32
	Levofloxacin	>32	>32	>32	>32	>32	>32	>32	>32
	Moxifloxacin	32	16	>32	16	32	32	>32	16
Polymyxins	Colistin (Polymyxin E)	8	8	4	8	>32	16	16	16
	Polymyxin B	>32	>32	>32	>32	>32	>32	>32	>32
Combination	Azithromycin/Minocycline	S	S	S	S	S	S	S	S
	Azithromycin/Tigecycline	S	S	S	S	S	S	S	S
	Clarithromycin/Minocycline	S	S	S	S	S	S	S	S
	Clarithromycin/Tigecycline	S	S	S	S	S	S	S	S
	Minocycline/Tigecycline	S	S	S	S	S	S	S	S
	Doxycycline/Azithromycin	S	S	S	S	S	S	S	S
	Doxycycline/Clarithromycin	S	S	S	S	S	S	S	S
	Doxycycline/Tigecycline	S	S	S	S	S	S	S	S
Antifungal agents	Amphotericin B	4	8	8	8	8	8	4	4
	Voriconazole	2	4	2	2	4	4	8	4
	Itraconazole	2	2	1	1	2	2	4	4
	Fluconazole	2	4	2	2	4	2	4	8
	Anidulafungin	4	2	4	8	4	2	8	8
	Caspofungin	4	2	4	4	4	2	4	8
	Terbinafine	2	4	2	2	4	2	4	4
	Voriconazole/Terbinafine	I	I	I	I	I	I	I	I
	Itraconazole/Terbinafine	I	I	I	I	I	I	I	I

The minimum inhibitory concentrations (MICs) of each agent were determined by 100% inhibition of mycelium by visual observation, comparted to the inhibition in the control wells (without antibiotics). S: synergism; I: indifference.

## Data Availability

The data presented in this study cannot be shared due to ethical, legal, and confidential issues in accordance with consent provided by participants.
